# ﻿Ambusher in sponge: a new species of *Eunice* (Annelida, Eunicidae) commensal within deep-sea Farreidae (Porifera, Hexactinellida) on northwest Pacific seamounts

**DOI:** 10.3897/zookeys.1230.140329

**Published:** 2025-03-05

**Authors:** Yadong Zhou, Ruiyan Zhang, Chengcheng Shen, Qin Mao, Mouyingke Zhang, Dongsheng Zhang

**Affiliations:** 1 Key Laboratory of Marine Ecosystem Dynamics, Second Institute of Oceanography, Ministry of Natural Resources, Hangzhou, Zhejiang, China Second Institute of Oceanography, Ministry of Natural Resources Hangzhou China; 2 Southern Marine Science and Engineering Guangdong Laboratory (Zhuhai), Zhuhai Guangdong, China Southern Marine Science and Engineering Guangdong Laboratory (Zhuhai) Zhuhai Guangdong China; 3 School of Oceanography, Shanghai Jiao Tong University, Shanghai, China Shanghai Jiao Tong University Shanghai China

**Keywords:** Gut content, morphology, Polychaeta, sponge associated species, taxonomy

## Abstract

Deep-sea sponges create complex biogenic structures and attract a wide array of deep-sea organisms, including symbionts. In this study, we describe *Eunicesiphoninsidiator***sp. nov.**, a new eunicid species living in the central cavity of deep-sea farreid glass sponges found on northwest Pacific seamounts. The new species closely resembles the Atlantic *Eunicenorvegica* both morphologically and molecularly, but it differs in the relative length of palp compared to peristomium, starting points of subacicular hooks, and shape of pectinate chaetae. A 13% *COI* genetic distance between the two species further supports the establishment of *E.siphoninsidiator* as a distinct species. Gut content analyses reveals fragments of barnacles and brittle stars, suggesting a carnivorous diet and a sit-and-wait predatory strategy. The eunicid gains protection from living inside the sponge, which consistently harbored the polychaete in all specimens examined, while the sponge benefits from the cleaning of epibionts, pointing to a potentially mutualistic relationship.

## ﻿Introduction

*Eunice* Cuvier, 1817 is a diverse genus with more than 200 currently accepted species (Read and Fauchald 2024) occurring worldwide in a wide variety of marine habitats, from shallow water to deep sea and including, among others, soft sediments, rocky bottoms, sponge conglomerates, coral rubble, and deep-sea hydrothermal vents (Hutchings 1986; Fauchald 1992; Desbruyères et al. 2006; Martin and Britayev 2018; Zanol et al. 2020). However, the associations involving species of *Eunice* and sponges are exclusively known from shallow waters (Zanol et al. 2021). In the nutrient-limited deep sea, glass sponges are important habitat modifiers, which increase the three-dimensional habitat complexity and attract a wide variety of organisms (Maldonado et al. 2015; Hanz et al. 2022).

Numerous species of polychaetes are known to live in association with these deep-sea sponges, but a few belong to *Eunice*. Among them, there are *Eunicegoodei* Fauchald, 1992 (as *Nicidionkinbergi* Webster), *Eunicedenticulata* Webster, 1884 (as *Leodicedenticulata* Webster), and *Eunicespongicola* (Treadwell, 1921) (as *Leodicespongicola* Treadwell, 1921) (Treadwell 1921), all of them found in soft bottoms of the Caribbean Sea (Treadwell 1921). Notably, this study reports and formally describes new species of *Eunice* living as a commensal of glass sponges (Porifera, Farreidae) found on deep-sea seamounts in the northwest Pacific. In addition to describing the characteristics of the association, we have analyzed the gut contents of the worms to uncover their potential food sources to support defining the type of association with its host sponges. These results expand our view of sponge-dwelling biodiversity in the largely unknown deep sea.

## ﻿Material and methods

### ﻿Sample collection, treatment, and observation

**Figure 1.**
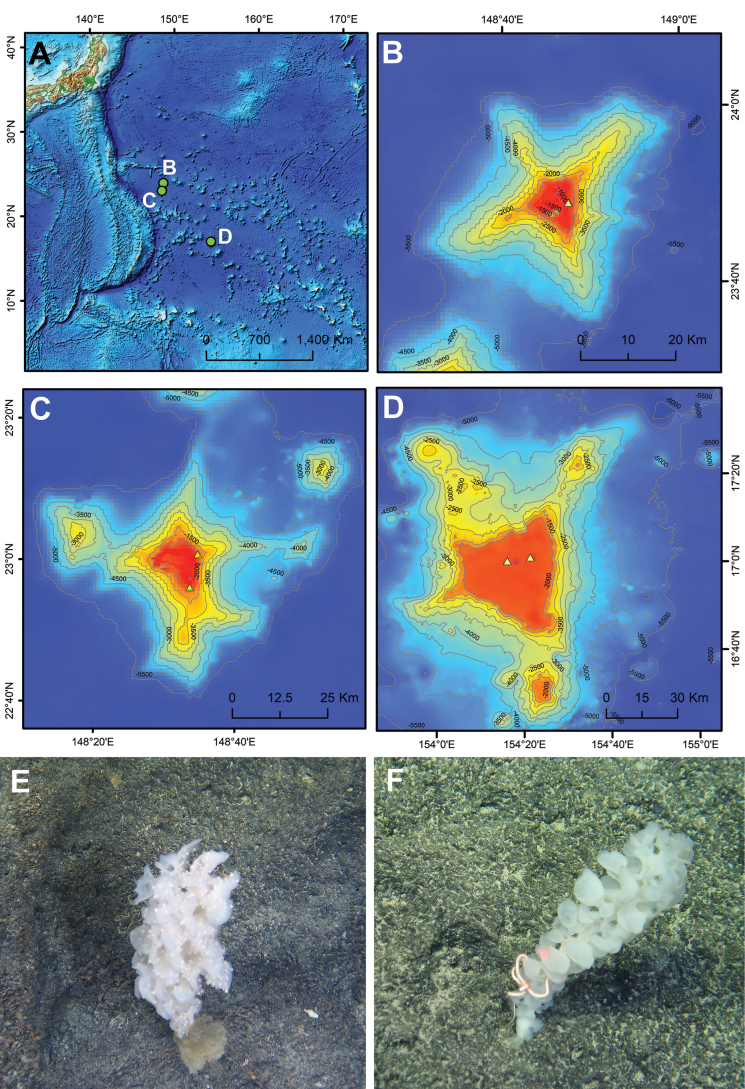
Map of sampling locations (**A–D**) and *in situ* images of farreid glass sponges captured during the *Jiaolong* dive JL230 (**E**) and JL233 (**F**) **B** DD Seamount **C** O-Hakucho Guyot **D** Albo Guyot.

**Figure 2.**
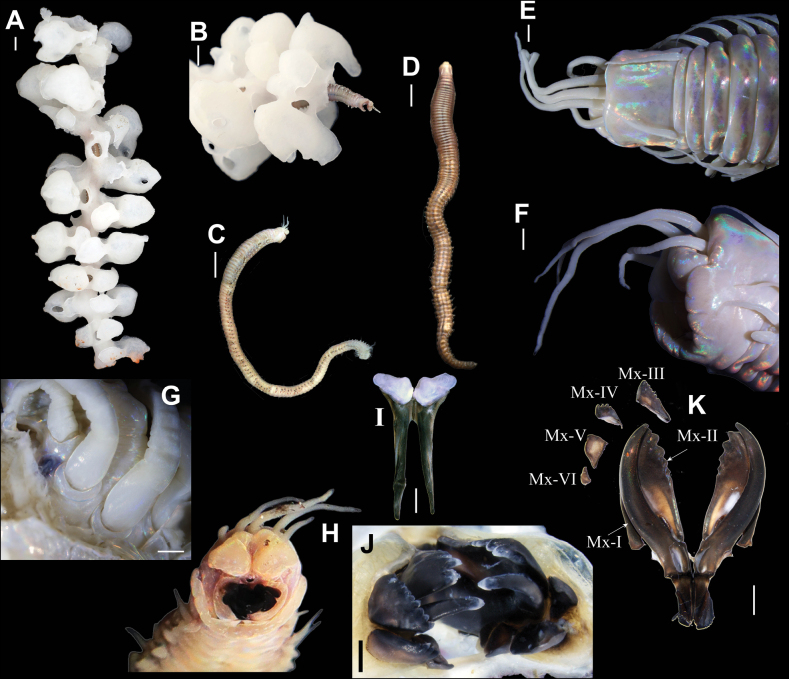
*Eunicesiphoninsidiator* sp. nov. **A** paratype (RSIOPOLY80002) commensal within a farreid sponge collected during the dive JL233 **B** anterior part of paratype (RSIOPOLY80002), anterior-ventral view **C** paratype (RSIOPOLY80002, before fixation), lateral view **D** holotype (RSIOPOLY80001, before fixation), dorsal view **E** anterior end of holotype, dorsal view (fixed in 100% (v/v) ethanol) **F** detail of anterior end of holotype, lateral view (fixed in 100% (v/v) ethanol) **G** location of eye in the paratype, dorsal view (fixed in 100% (v/v) ethanol) **H** anterior end of paratype, frontal view (fixed in 100% ethanol) **I** mandible of paratype, ventral view **J** maxillae apparatus of paratype (RSIOPOLY80002), frontal view (before digestion with Proteinase K) **K** maxillae apparatus of paratype (RSIOPOLY80002), dorsal view (after digestion with Proteinase K). Scale bars: 1 cm (**A–D**); 1 mm (**E, F**); 0.5 mm (**G, J**); 1 mm (**I, K**).

Seven farreid sponges were collected from O-Hakucho Guyot, Albo Guyot; DD seamount (Fig. 1), using the manipulator of the human-occupied vehicle (HOV) *Jiaolong* during cruises DY80 and DY86 onboard R/V *Shenhaiyihao*. Onboard, single eunicids were picked out from the central cavity of six sponges, while two specimens were extracted from the seventh sponge (RSIOPOLY86001 and RSIOPOLY86003) (Fig. 2A, B). All specimens were preserved in 100% (v/v) ethanol prior to being observed and photographed under a Zeiss Discovery V.16/V.20 stereomicroscope. The maxillary apparatus and parapodia were dissected and examined under a stereomicroscope (Carl Zeiss SteREO Discovery V.20). Selected parapodia from anterior, middle, and posterior regions were dissected and prepared for morphological examinations of dorsal cirri, ventral cirri, and chaetae under a microscope (Carl Zeiss Imager. A2). Two specimens were dissected to extract the gut contents, which were then examined under a stereomicroscope. Undigested tissues were picked out, photographed, and analyzed to determine their potential origins.

Type specimens and material examined are deposited at the repository of the Second Institute of Oceanography (**RSIO**), Ministry of Natural Resources, Hangzhou, China.

### ﻿Molecular analysis

Total genomic DNA was extracted from a dissected parapodium using the Mollusc DNA kit (Omega, USA) following manufacturer instructions. Gene fragments of the cytochrome *c* oxidase subunit I gene (COI) were amplified using the primer pairs HCO2198/LCO1490 for (Folmer et al. 1994), following the thermal program 95 °C for 4 min, 35 cycles of 95 °C for 30 s, 45 °C for 45 s, and 72 °C for 1 min, and 72 °C for 7 min. Purifications and bidirectional Sanger sequencing of the polymerase chain reaction (PCR) products were then conducted in Sangon (Shanghai China). Consensus sequences (ca 660 bp) were assembled in Geneious R11 after examination of the raw sequences by eye (Kearse et al. 2012). A *COI* dataset (Suppl. material 1), including the newly generated and publicly available sequences, was created and aligned using the MUSCLE algorithm (Edgar 2004) within Geneious R11. The result was then used to estimate the genetic distances between the new species and its congeners in MEGA v. 11 (Tamura et al. 2021), with the Kimura 2-parameters (K2P) substitution model (Suppl. material 2).

## ﻿Results

### ﻿Systematics

**Eunicidae Berthold, 1827**

***Eunice* Cuvier, 1817**

#### ﻿ Eunice siphoninsidiator

Taxon classificationAnimaliaEunicidaEunicidae

Zhou, Zhang, Shen & Zhang sp. nov.

93864158-DB3A-51CC-88C3-A5862B63C33F

https://zoobank.org/3351F071-3A6F-483E-8591-1102FE2FA028

Figs 2 , 3

##### Type material.

***Holotype***: • RSIOPOLY80001, O-Hakucho Guyot, Northwest Pacific, 23.01°N, 148.58°E, 1135 m depth; *Jiaolong* dive JL230, R/V *Shenhaiyihao* cruise DY80-I; 27 June 2023; fixed in 100% (v/v) ethanol. ***Paratype***: • two specimens. Paratype 1#, RSIOPOLY80002, O-Hakucho Guyot, Northwest Pacific, 22.93°N, 148.56°E, 1175 m depth; *Jiaolong* dive JL233, R/V *Shenhaiyihao* cruise DY80-I; 30 June 2023; fixed in 100% (v/v) ethanol; • ***Paratype*** 2#, RSIOPOLY86005, Albo Guyot, Northwest Pacific, 17.0001°N, 154.2679°E, 1057 m depth, *Jiaolong* dive JL311, R/V *Shenhaiyihao* cruise DY86-II, 3 September 2024, fixed in 10% (v/v) formalin, partial tissue fixed in 100% (v/v) ethanol.

##### Additional material.

Five specimens, incomplete. • RSIOPOLY86001 and RSIOPOLY86003, Albo Guyot, Northwest Pacific, 16.9998°N, 154.7687°E, 1107 m depth, *Jiaolong* dive JL311, R/V *Shenhaiyihao* cruise DY86-II, 3 September 2024, anterior part fixed in 100% (v/v) ethanol; • RSIOPOLY86014, DD Seamount, Northwest Pacific, 23.8150°N, 148.7926°E, 1055 m depth, *Jiaolong* dive JL302, R/V *Shenhaiyihao* cruise DY86-II, 20 August 2024, anterior part fixed in 100% (v/v) ethanol; • RSIOPOLY86015, DD Seamount, Northwest Pacific, 23.8150°N, 148.7915°E, 1047 m depth, *Jiaolong* dive JL302, R/V *Shenhaiyihao* cruise DY86-II, 20 August 2024, anterior part fixed in 100% (v/v) ethanol; • RSIOPOLY86016, Albo Guyot, Northwest Pacific, 17.0143°N, 154.3549°E, 1126 m depth, *Jiaolong* dive JL312, R/V *Shenhaiyihao* cruise DY86-II, 4 September 2024, anterior part fixed in 100% (v/v) ethanol.

##### Measurements

**(before fixation).** Holotype complete, with 95 chaetigers, total length 177 mm, first 10 chaetigers 11.5 mm in length, width at chaetiger 10 without parapodia 10 mm. Paratype 1# complete, with 107 chaetigers, total length 140 mm, first 10 chaetigers 8.1 mm in length, width at chaetiger 10 without parapodia 6.1 mm. Paratype 2# complete, with 103 chaetigers, total length 151 mm, first 10 chaetigers 11.2 mm in length, width at chaetiger 10 without parapodia 6.7 mm.

##### Description.

Live specimens iridescent brownish or slightly pinkish with lighter patches along the body. Preserved specimens pale white, slightly iridescent. Body long, dorsally convex, and ventrally flat (Fig. 2C, D).

Prostomium narrower and shorter than first peristomial ring, bilobed anteriorly with round anterior ends and deep median sulcus (Fig. 2E, F). Prostomial appendages in semicircle, with lateral antennae closer to palps than to median antennae (Fig. 2E–H). Antennae 2 × longer than palps, reaching anterior end of chaetiger 5 when reversing; median antennae about 1.5 × as long as lateral antennae (Fig. 2E, F). Ceratophores of antennae and palps short and ring-shaped, usually covered by prostomium; ceratostyles long, tapering, with indistinct articulations; palpstyles tapering digitiform distally with indistinct articulations (Fig. 2E–G). Eyes dark, lateral to ceratophores of lateral antennae (Fig. 2G).

Peristomium cylindrical, separation between first and second ring visible on both dorsal and ventral sides (Fig. 2E, F); first ring more than 5/6 as long as of whole peristomium; peristomial cirri tapering, indistinctly articulated, reaching anterior end (middle in paratype) of first peristomial ring (Fig. 2E, F).

Mandibles dark brown, with white wing-shaped calcareous cutting edges (Fig. 2I). Maxillary apparatus brown to dark brown. Maxillae formula MxI 1+1, MxII 6+5, MxIII 8+0, MxIV 5+5, MxV 1+1, MxVI 1+1. All teeth with blunt ends. Mx-I about 3 × as long as carrier. MxV with a large square ridge; MxVI reduced to a plate with a minute tooth (Fig. 2J, K).

**Figure 3.**
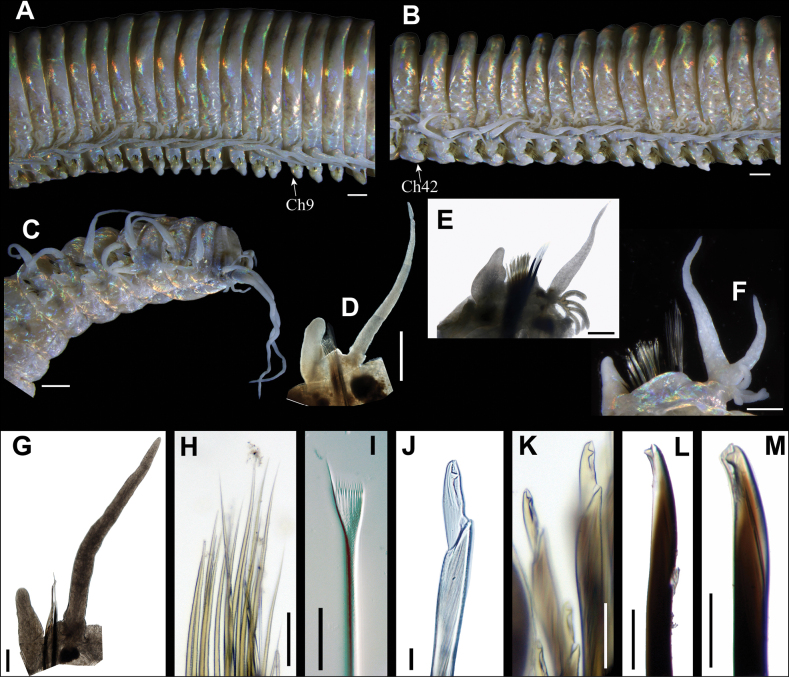
*Eunicesiphoninsidiator* sp. nov. **A** middle frontal segments of holotype, lateral view (right side) **B** middle segments of paratype in lateral view (right side) **C** posterior end of holotype, lateral view (left side) **D** right parapodium of chaetiger 4 of paratype (RSIOPOLY86005, frontal view) **E** right parapodium of chaetiger 30 of holotype (frontal view) **F** right parapodium of chaetiger 44 of holotype (frontal view) **G** right parapodium of chaetiger 103 of paratype (RSIOPOLY86005, frontal view) **H** limbate chaetae on chaetiger 30 **I** pectinate chaetae on chaetiger 64 of paratype (RSIOPOLY86005) **J** compound falcigers on chaetiger 54 of paratype (RSIOPOLY80002) **K** compound falcigers on chaetiger 30 of paratype (RSIOPOLY80002) **L** subacicular hooks on a posterior chaetiger of paratype (RSIOPOLY80002) **M** subacicular hooks on chaetiger 50 of paratype (RSIOPOLY80002). Scale bars: 1 mm (**A–D**); 0.5 mm (**E, F**); 0.2 mm (**G**); 0.1 mm (**H, J–L**); 50 μm (**I**).

Branchiae from chaetiger 9 to near posterior end (Fig. 3A–C), pectinate, always shorter and slender than dorsal cirri (Fig. 3A, B), with 1–2 short filaments on most anterior and most posterior branchial segments, increasing to 4–5 filaments on following parapodia and reaching a maximum of 7–8 at middle region (Fig. 3D–F).

Dorsal cirri tapering, indistinctly articulated distally in anterior chaetigers, then smooth, slightly thicker on prebranchial segments and slender on posterior segments (Fig. 3A–G). Ventral cirri 1/4–1/3 and 2–3 as long as dorsal cirri and chaetal lobes, respectively, gradually shortening from anterior to posterior regions. First four ventral cirri short, digitiform, strong; following ones with suboval basal inflation and a short tapering tip from chaetiger 5 through 37; subsequent ventral cirri digitiform (Fig. 3D–G).

Chaetal lobs obliquely truncate, longer dorsally than ventrally, with aciculae emerging dorsal to midline. Prechaetal lobe low transverse fold. Postchaetal lobes round or obliquely truncate, inflated on all chaetigers. Limbate chaetae supracicular, tapering and slender (Fig. 3 H). Thin pectinate chaetae emerging from chaetiger 5 to last chaetiger, tapering, flat with 12–16 teeth; one marginal teeth longer than others (Fig. 3I). Shafts of compound falcigers smooth, slender at basal part, expanded distally; blades bidentate, with proximal tooth triangular, similar to distal tooth in length, directed laterally; distal tooth slightly curved, slightly slenderer than proximal tooth (Fig. 3J, K). Aciculae dark brown, straight or slightly bent, tapering with blunt or pointed tips, 1–3 in each parapodium (Fig. 3E). Subacicular hook bidentate, dark brown, from chaetiger 27 to last segment, 1–2 per parapodium; proximal tooth triangular, directed laterally; distal tooth, smaller than proximal tooth, directed upwards (Fig. 3L, M).

Pygidium with two pairs of smooth anal cirri, most ventral pair short and slender; dorsal pair much longer and thicker, extending to last 6^th^ chaetiger when folded anteriorly (Fig. 3C).

##### Variation.

Relative length of peristomial cirri to first ring of peristomium close to 1:1 in holotype, paratype 2# and 4 other individuals, but close to 1:2 in paratype 1#. Holotype with bifurcated dorsal cirri in left parapodia of chaetigers 14 and 45 and in right parapodia of chaetiger 44, absent in paratype (Fig. 3B, F). Starting of branchiae and subacicular hook ranging from chaetiger 8–11 and 27–32, respectively, in all examined specimens.

##### Etymology.

A combination of two Latin words: ‘*siphon*-’ meaning ‘tube’ or ‘pipe’ and ‘*insidiator*’ meaning ‘ambusher’. This is a reference to the behavior of the species as ambush predator living within tubular structure of glass sponges. This name is to be treated as noun in apposition.

##### Distribution.

Found living in association with deep-sea glass sponges on seamounts in the northwest Pacific.

##### Remarks.

*Eunicesiphoninsidiator* sp. nov. belongs to group B-2 as proposed by Fauchald (1992) based on its dark bidentate subacicular hooks and high percentage of branchial chaetigers (>65%). It has indistinctly articulated prostomial appendages and anterior dorsal cirri, and reduced branchiae with only a few short branchial filaments (<8 and shorter than the notopodial dorsal cirri), thus most closely resembles *Eunicenorvegica* (Linnaeus, 1767). However, *E.siphoninsidiator* sp. nov. has a ~1/1 relative length of palps compared to peristomium (1/2 in *E.norvegica*), subacicular hooks starting from chaetiger 27–32 (42/44 in *E.norvegica*) and pectinate chaetae asymmetric, with one marginal tooth much longer than others (symmetric, with two slightly longer marginal teeth in *E.norvegica*). Moreover, they differ by 13% in K2P genetic distance (Suppl. material 2).

### ﻿Gut content analyses

**Figure 4.**
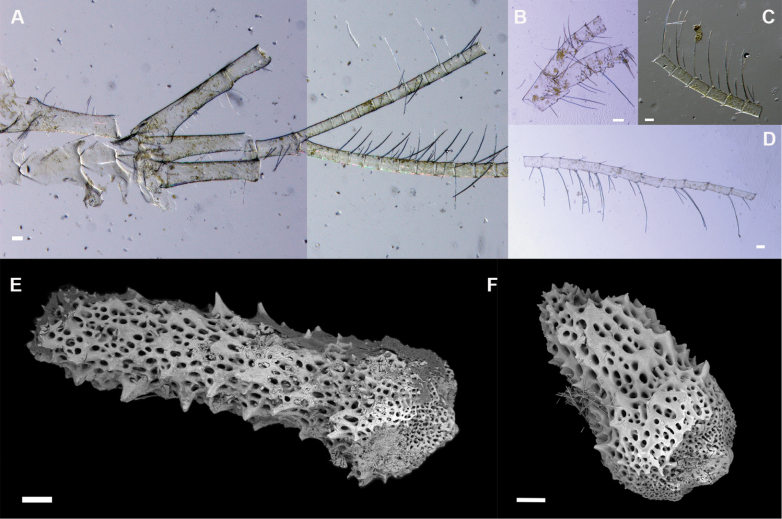
Gut contents of paratype **A–D** fragments of stalked-barnacle ctenopod cirri **E, F** fragments as brittle star arm spines. Scale bars: 0.1 mm (**A–F**).

It was not possible to identify gut content fragments to species level, but some were ctenopod cirri of stalked-barnacles and arm spines of brittle stars (Fig. 4).

## ﻿Discussion

Habitat forming species (HFS) living in the food-limited deep sea, such as sponges and corals, significantly enhance small-scale environmental heterogeneity, providing various ecological functions for other animals, such as protection from predation, refugia, nursery habitats and feeding grounds (De La Torriente et al. 2020). A wide variety of animals have been found to be associated with deep-sea sponges, including fish, echinoderms, crustaceans, and polychaetes, the latter mainly represented by polynoids and syllids and more rarely by eunicids (Martin and Britayev 1998, 2018; Maldonado et al. 2015; Taboada et al. 2021).

Among eunicids, *Eunice* species are considered either as free-living (Jumars et al. 2015) or as symbionts, which are associated with cnidarians (including deep-sea cold-water corals), gastropods, echiurids, and shallow-water sponges (Treadwell 1921; Fauchald 1992; Martin and Britayev 1998, 2018). Therefore, this study marks the first documented case of a eunicid inhabiting deep-sea sponges.

Symbiotic eunicids were initially regarded as commensals or inquilines, obtaining shelter from their hosts (Martin and Britayev 1998). However, mutualistic characters were revealed in the association between *E.norvegica* and the cold-water reef-coral *Desmophyllumpertusum* (Linnaeus, 1758). The eunicid contributes to reef-patch formation by connecting coral fragments, to reef cleaning by active sediment transport and to protect host coral from sea urchin’s predation (Mortensen 2001; Roberts 2005; Mueller et al. 2013). In return, the eunicid obtains more available food either thanks to the water flow produced by the hosts or by stealing food captured by the hosts (Mortensen 2001). Thus, we speculate that *E.siphoninsidiator* sp. nov. might not only use its farreid host as a shelter but also avail the host’s water circulation for feeding.

Moreover, *E.siphoninsidiator* sp. nov. seems to obtain additional food from species “attracted” by the refuge provided by its host, as glass sponges are key HFS in the barren deep sea. This increases possibilities of inter- and intra-specific interactions, including predation. Thus, we cannot discard *E.siphoninsidiator* sp. nov. behaving as a sit-and-wait predator. Its gut content contained remains of barnacles and brittle stars, which inhabit the sponge outer surface to elevate themselves off the seafloor and improve their filtering efficiency. Inadvertently, this places themselves within reach of the ambushing worm hidden inside the sponge, which may obtain prey while avoiding being predated. Such a feeding strategy may also play a key role in cleaning the sponge from unwanted epibionts, thereby supporting the sponge’s filtration efficiency. This potentially reciprocal benefit suggests a mutualistic relationship, which appears to be obligate, as all farreid sponges examined hosted specimens of *E.siphoninsidiator* sp. nov. Nevertheless, further research is needed to confirm the nature of this interaction and the extent of its ecological implications.

## Supplementary Material

XML Treatment for Eunice siphoninsidiator

## ﻿Additional information

### Author ORCIDs

Yadong Zhou https://orcid.org/0000-0002-3577-1118

Ruiyan Zhang https://orcid.org/0000-0001-8556-8145

Chengcheng Shen https://orcid.org/0000-0002-2505-0974

Dongsheng Zhang https://orcid.org/0000-0002-2736-3229

### Data availability

Molecular data of the new species is deposited in NCBI GenBank, with the accession numbers PQ517215-PQ517222.

## Supplementary materials

Supplementary material 1 *COI* sequences used for genetic distance calculation Data type docx Explanation note Species and accession numbers of *COI* sequences used for genetic distance calculation. https://binary.pensoft.net/file/1269940This dataset is made available under the Open Database License (http://opendatacommons.org/licenses/odbl/1.0/). The Open Database License (ODbL) is a license agreement intended to allow users to freely share, modify, and use this Dataset while maintaining this same freedom for others, provided that the original source and author(s) are credited.Yadong Zhou, Ruiyan Zhang, Chengcheng Shen, Qin Mao, Mouyingke Zhang, Dongsheng Zhang

Supplementary material 2 Pairwise K2P distance (based on partial *COI* sequences) between known *Eunice* species with available DNA data Data type docx https://binary.pensoft.net/file/1269941This dataset is made available under the Open Database License (http://opendatacommons.org/licenses/odbl/1.0/). The Open Database License (ODbL) is a license agreement intended to allow users to freely share, modify, and use this Dataset while maintaining this same freedom for others, provided that the original source and author(s) are credited.Yadong Zhou, Ruiyan Zhang, Chengcheng Shen, Qin Mao, Mouyingke Zhang, Dongsheng Zhang
