# Supplementary material for: ﻿Ambusher in sponge: a new species of Eunice (Annelida, Eunicidae) commensal within deep-sea Farreidae (Porifera, Hexactinellida) on northwest Pacific seamounts
Source: Zookeys. 2025 Mar 5;1230:25–36. doi: 10.3897/zookeys.1230.140329 (PMC11904825; doi:10.3897/zookeys.1230.140329)
Supplement: Supplementary material 1 — COI sequences used for genetic distance calculation [file zookeys-1230-025_article-140329__-s001.docx]

Table S1 *COI* sequences used for genetic distance calculation.

| No. | Species | Accession number |
| --- | --- | --- |
| 1 | *E. antennata* | KM094193 MH337357 |
| 2 | *E*. aff. *antennata* HAW01 | MW278379 |
| 3 | *E*. aff. *antennata* HAW02 | MW277779 MW277804 MW277870 MW277871 MW277973 MW277977 MW278018 MW278288 MW278482 MW278560 MW278801 |
| 4 | *E. afra* | MW278016 MW278401 |
| 5 | *E. americana* | GQ497561 |
| 6 | *E. amoureuxi* | GQ497538 |
| 7 | *E. antarctica* | GQ497532 |
| 8 | *E. aphroditois* | MN690243 |
| 9 | *E.* cf. *aphroditois* | LC756626 LC756629 LC756636 LC756634 LC756627 LC756623 LC756624 LC756625 LC756631 LC756632 LC756633 LC756628 LC756635 LC756630 |
| 10 | *E*. cf. *antillensis* | GQ497533 |
| 11 | *E.* cf. *insularis* | GQ497537 |
| 12 | *E*. cf. *violaceomaculata* | GQ497542 |
| 13 | *E. filamentosa* | GQ497545 |
| 14 | *E. indica* | MN256537 MN256538 |
| 15 | *E. lucei* | GQ497529 |
| 16 | *E. miurai* | GQ497530 |
| 17 | *E. mutilata* | GQ497540 |
| 18 | *E. norvegica* | MN826130 GQ497541 KT592274 |
| 19 | *E. notata* | GQ497544 |
| 20 | *E. roussaei* | OL889810 OL889805 OL889816 OL889808 OL889811 GQ497543 OL889818 OL889807 OL889819 OL889813 OL889814 OL889820 OL889815 OL889821 OL889806 OL889809 OL889812 OL889817 OL889804 |
| 21 | *E. rubra* | GQ497528 |
| 22 | *E. samoae* | JX559752 |
| 23 | *E. siphoninsidiator* sp. nov. | PQ517215 PQ517216 PQ517217 PQ517218 PQ517219 PQ517220 PQ517221 PQ517222 |
| 24 | *E. thomasiana* | GQ497563 |
| 25 | *E. torquata* | GQ497539 |
| 26 | *E. tubifex* | MH337354 |
| 27 | *E. vittata* | KT307642 ON716077 ON716087 ON716097 |
