# Supplementary material for: ﻿Ambusher in sponge: a new species of Eunice (Annelida, Eunicidae) commensal within deep-sea Farreidae (Porifera, Hexactinellida) on northwest Pacific seamounts
Source: Zookeys. 2025 Mar 5;1230:25–36. doi: 10.3897/zookeys.1230.140329 (PMC11904825; doi:10.3897/zookeys.1230.140329)
Supplement: Supplementary material 2 — Pairwise K2P distance (based on partial COI sequences) between known Eunice species with available DNA data [file zookeys-1230-025_article-140329__-s002.docx]

Table S2 Pairwise K2P distance (based on partial COI sequences) between known *Eunice* species with available DNA data.

|  |  | 1 | 2 | 3 | 4 | 5 | 6 | 7 | 8 | 9 | 10 | 11 | 12 | 13 |
| --- | --- | --- | --- | --- | --- | --- | --- | --- | --- | --- | --- | --- | --- | --- |
| 1 | *E. antennata* | 0.0000 |  |  |  |  |  |  |  |  |  |  |  |  |
| 2 | *E.* aff*. antennata* HAW01 | 0.3032 | -- |  |  |  |  |  |  |  |  |  |  |  |
| 3 | *E.* aff*. antennata* HAW02 | 0.3479 | 0.2272 | 0.0000 |  |  |  |  |  |  |  |  |  |  |
| 4 | *E. afra* | 0.3523 | 0.3439 | 0.3567 | 0.0033 |  |  |  |  |  |  |  |  |  |
| 5 | *E. americana* | 0.3052 | 0.2781 | 0.3288 | 0.3656 | -- |  |  |  |  |  |  |  |  |
| 6 | *E. amoureuxi* | 0.5093 | 0.4865 | 0.4622 | 0.3161 | 0.4761 | -- |  |  |  |  |  |  |  |
| 7 | *E. antarctica* | 0.3298 | 0.2502 | 0.3467 | 0.3340 | 0.2169 | 0.4268 | -- |  |  |  |  |  |  |
| 8 | *E. aphroditois* | 0.4421 | 0.3207 | 0.3713 | 0.2486 | 0.3381 | 0.3639 | 0.3331 | -- |  |  |  |  |  |
| 9 | *E.* cf*. aphroditois* | 0.3902 | 0.3102 | 0.4291 | 0.2864 | 0.3320 | 0.3757 | 0.3176 | 0.1104 | 0.0070 |  |  |  |  |
| 10 | *E.* cf*. antillensis* | 0.3665 | 0.2920 | 0.3516 | 0.3682 | 0.3159 | 0.4609 | 0.3223 | 0.3094 | 0.3072 | -- |  |  |  |
| 11 | *E.* cf*. insularis* | 0.4278 | 0.4167 | 0.4654 | 0.3446 | 0.4629 | 0.3912 | 0.3904 | 0.3183 | 0.3434 | 0.4149 | -- |  |  |
| 12 | *E.* cf*. violaceomaculata* | 0.3661 | 0.3645 | 0.4004 | 0.2760 | 0.3941 | 0.3943 | 0.3074 | 0.2582 | 0.2608 | 0.2799 | 0.3510 | -- |  |
| 13 | *E. filamentosa* | 0.3695 | 0.3020 | 0.3989 | 0.2816 | 0.3813 | 0.3828 | 0.3384 | 0.2591 | 0.2860 | 0.2948 | 0.3195 | 0.2270 | -- |
| 14 | *E. indica* | 0.4810 | 0.4348 | 0.4348 | 0.4025 | 0.4546 | 0.4359 | 0.3734 | 0.3421 | 0.3899 | 0.4234 | 0.3973 | 0.3454 | 0.3837 |
| 15 | *E. lucei* | 0.2352 | 0.2782 | 0.3306 | 0.3838 | 0.3131 | 0.4657 | 0.2987 | 0.3561 | 0.3403 | 0.3350 | 0.4310 | 0.4308 | 0.3559 |
| 16 | *E. miurai* | 0.3145 | 0.3148 | 0.3493 | 0.4376 | 0.3557 | 0.4646 | 0.3263 | 0.4037 | 0.3805 | 0.3697 | 0.4748 | 0.4109 | 0.4195 |
| 17 | *E. mutilata* | 0.4993 | 0.4837 | 0.4492 | 0.3066 | 0.4299 | 0.0289 | 0.4121 | 0.3553 | 0.3733 | 0.4636 | 0.3786 | 0.3731 | 0.4098 |
| 18 | *E. norvegica* | 0.4092 | 0.4089 | 0.4872 | 0.3291 | 0.4154 | 0.3957 | 0.3856 | 0.3019 | 0.2802 | 0.3642 | 0.4368 | 0.2988 | 0.3177 |
| 19 | *E. notata* | 0.3833 | 0.3160 | 0.3512 | 0.1656 | 0.3748 | 0.3273 | 0.3070 | 0.2753 | 0.3202 | 0.3297 | 0.3282 | 0.2782 | 0.2788 |
| 20 | *E. roussaei* | 0.3980 | 0.3390 | 0.4009 | 0.3359 | 0.3250 | 0.4142 | 0.3193 | 0.2651 | 0.2853 | 0.3578 | 0.3431 | 0.2383 | 0.2852 |
| 21 | *E. rubra* | 0.0884 | 0.2763 | 0.3338 | 0.3517 | 0.2738 | 0.5216 | 0.2990 | 0.4258 | 0.4037 | 0.3301 | 0.3985 | 0.3732 | 0.3430 |
| 22 | *E. samoae* | 0.4775 | 0.4928 | 0.5473 | 0.3684 | 0.4394 | 0.2404 | 0.4470 | 0.3700 | 0.3655 | 0.4649 | 0.3287 | 0.3887 | 0.3842 |
| 23 | ***E. siphoninsidiator* sp. nov.** | 0.4070 | 0.3292 | 0.4351 | 0.2969 | 0.3665 | 0.3933 | 0.3370 | 0.2659 | 0.2763 | 0.3510 | 0.3660 | 0.2706 | 0.3187 |
| 24 | *E. thomasiana* | 0.3719 | 0.3093 | 0.3068 | 0.3271 | 0.2819 | 0.3946 | 0.3009 | 0.2651 | 0.2955 | 0.2705 | 0.3199 | 0.2639 | 0.2584 |
| 25 | *E. torquata* | 0.3129 | 0.3090 | 0.3462 | 0.2753 | 0.2874 | 0.3553 | 0.2762 | 0.2828 | 0.2971 | 0.2470 | 0.3259 | 0.2578 | 0.2799 |
| 26 | *E. tubifex* | 0.5415 | 0.4998 | 0.6089 | 0.5602 | 0.5260 | 0.5792 | 0.5629 | 0.5164 | 0.5678 | 0.5434 | 0.6043 | 0.5625 | 0.4839 |
| 27 | *E. vittata* | 0.3531 | 0.2969 | 0.3182 | 0.3824 | 0.3140 | 0.4623 | 0.3479 | 0.3903 | 0.4128 | 0.3620 | 0.4389 | 0.4043 | 0.3875 |

Table 2 (Continued)

|  |  | 14 | 15 | 16 | 17 | 18 | 19 | 20 | 21 | 22 | 23 | 24 | 25 | 26 | 27 |
| --- | --- | --- | --- | --- | --- | --- | --- | --- | --- | --- | --- | --- | --- | --- | --- |
| 1 | *E. antennata* |  |  |  |  |  |  |  |  |  |  |  |  |  |  |
| 2 | *E.* aff*. antennata* HAW01 |  |  |  |  |  |  |  |  |  |  |  |  |  |  |
| 3 | *E.* aff*. antennata* HAW02 |  |  |  |  |  |  |  |  |  |  |  |  |  |  |
| 4 | *E. afra* |  |  |  |  |  |  |  |  |  |  |  |  |  |  |
| 5 | *E. americana* |  |  |  |  |  |  |  |  |  |  |  |  |  |  |
| 6 | *E. amoureuxi* |  |  |  |  |  |  |  |  |  |  |  |  |  |  |
| 7 | *E. antarctica* |  |  |  |  |  |  |  |  |  |  |  |  |  |  |
| 8 | *E. aphroditois* |  |  |  |  |  |  |  |  |  |  |  |  |  |  |
| 9 | *E.* cf*. aphroditois* |  |  |  |  |  |  |  |  |  |  |  |  |  |  |
| 10 | *E.* cf*. antillensis* |  |  |  |  |  |  |  |  |  |  |  |  |  |  |
| 11 | *E.* cf*. insularis* |  |  |  |  |  |  |  |  |  |  |  |  |  |  |
| 12 | *E.* cf*. violaceomaculata* |  |  |  |  |  |  |  |  |  |  |  |  |  |  |
| 13 | *E. filamentosa* |  |  |  |  |  |  |  |  |  |  |  |  |  |  |
| 14 | *E. indica* | 0.0000 |  |  |  |  |  |  |  |  |  |  |  |  |  |
| 15 | *E. lucei* | 0.4195 | -- |  |  |  |  |  |  |  |  |  |  |  |  |
| 16 | *E. miurai* | 0.4395 | 0.2535 | -- |  |  |  |  |  |  |  |  |  |  |  |
| 17 | *E. mutilata* | 0.4094 | 0.4625 | 0.4544 | -- |  |  |  |  |  |  |  |  |  |  |
| 18 | *E. norvegica* | 0.4074 | 0.4554 | 0.4403 | 0.3935 | 0.0161 |  |  |  |  |  |  |  |  |  |
| 19 | *E. notata* | 0.3705 | 0.4271 | 0.4026 | 0.3482 | 0.3667 | -- |  |  |  |  |  |  |  |  |
| 20 | *E. roussaei* | 0.4059 | 0.3907 | 0.4120 | 0.4056 | 0.3973 | 0.3200 | 0.0069 |  |  |  |  |  |  |  |
| 21 | *E. rubra* | 0.4428 | 0.2336 | 0.3140 | 0.4799 | 0.4221 | 0.3696 | 0.3572 | -- |  |  |  |  |  |  |
| 22 | *E. samoae* | 0.4482 | 0.4446 | 0.5305 | 0.2272 | 0.4144 | 0.3326 | 0.4437 | 0.4637 | -- |  |  |  |  |  |
| 23 | ***E. siphoninsidiator* sp. nov.** | 0.3918 | 0.4246 | 0.4214 | 0.3905 | 0.1276 | 0.3023 | 0.3087 | 0.4170 | 0.3994 | 0.0000 |  |  |  |  |
| 24 | *E. thomasiana* | 0.3296 | 0.3073 | 0.3480 | 0.4099 | 0.3772 | 0.3145 | 0.3076 | 0.3271 | 0.3974 | 0.3207 | -- |  |  |  |
| 25 | *E. torquata* | 0.3264 | 0.3132 | 0.3442 | 0.3814 | 0.3489 | 0.2945 | 0.3447 | 0.2785 | 0.3926 | 0.3250 | 0.1782 | -- |  |  |
| 26 | *E. tubifex* | 0.5322 | 0.6356 | 0.5622 | 0.6209 | 0.5795 | 0.5247 | 0.4433 | 0.5844 | 0.6986 | 0.5280 | 0.5605 | 0.5763 | -- |  |
| 27 | *E. vittata* | 0.4278 | 0.3176 | 0.3905 | 0.4276 | 0.4512 | 0.3472 | 0.4104 | 0.3167 | 0.4727 | 0.3632 | 0.3189 | 0.3279 | 0.6067 | 0.0099 |
